# Anti-Biofilm Effects of Z102-E of *Lactiplantibacillus plantarum* against *Listeria monocytogenes* and the Mechanism Revealed by Transcriptomic Analysis

**DOI:** 10.3390/foods13162495

**Published:** 2024-08-08

**Authors:** Jinyuan Wei, Xingguo Zhang, Mohamedelfatieh Ismael, Qingping Zhong

**Affiliations:** Guangdong Provincial Key Laboratory of Food Quality and Safety, College of Food Science, South China Agricultural University, Guangzhou 510642, China; weijinyuan2021@163.com (J.W.); 13726138004@163.com (X.Z.); 2018071076@nwafu.edu.cn (M.I.)

**Keywords:** *Lactiplantibacillus plantarum*, *Listeria monocytogenes*, transcriptomic analysis, anti-biofilm, quorum sensing, motility

## Abstract

Lactic acid bacteria (LAB) are the most common probiotics, and they present excellent inhibitory effects on pathogenic bacteria. This study aimed to explore the anti-biofilm potential of the purified active substance of *Lactiplantibacillus plantarum*, named Z102-E. The effects of Z102-E on *Listeria monocytogenes* were investigated in detail, and a transcriptomic analysis was conducted to reveal the anti-biofilm mechanism. The results indicated that the sub-MIC of Z102-E (3.2, 1.6, and 0.8 mg/mL) decreased the bacterial growth and effectively reduced the self-aggregation, surface hydrophobicity, sugar utilization, motility, biofilm formation, AI-2 signal molecule, contents of extracellular polysaccharides, and extracellular protein of *L. monocytogenes*. Moreover, the inverted fluorescence microscopy observation confirmed the anti-biofilm effect of Z102-E. The transcriptomic analysis indicated that 117 genes were up-regulated and 214 were down-regulated. Z102-E regulated the expressions of genes related to *L. monocytogenes* quorum sensing, biofilm formation, etc. These findings suggested that Z102-E has great application potential as a natural bacteriostatic agent.

## 1. Introduction

*Listeria monocytogenes* is a Gram-positive bacterium that is non-spore-forming [1], resistant to various adverse conditions, and found ubiquitously in food and the environment, such as farms, fertilizers, soils, water sources, and air [1,2]. It has become one of the major microorganisms transmitted by dairy and meat products [3]. It is tenacious in its survival vigor and can grow and multiply under extreme conditions, such as −0.4 to 45 °C (optimally 30 to 37 °C), pH 4.0 to 9.6 (optimally 6 to 8), sodium chloride concentration greater than 10%, and water activity below 0.9 [1,4,5]. It causes listeriosis, leading to sepsis, encephalitis, meningitis, miscarriages, and stillbirths [6,7]. The mortality rate of this disease is relatively high (20~30%) [8,9], especially in immunocompromised patients, pregnant women and fetuses, neonates, and the elderly [10,11]. Rodriguez-Auad et al. found that the rate of *L. monocytogenes* infection in pregnant women is 13~20 times higher than that of ordinary people [2]. In recent years, food safety incidents triggered by *L. monocytogenes* have been increasing [12]. South Africa had a total of 1060 cases within the listeriosis outbreaks between 2017 and 2018, of which more than 400 were neonates, with a high case fatality rate of 28.6% [13]. In Europe, listeriosis is recognized as the second major cause of death from foodborne infection, and the number of incidents of *L. monocytogenes* infections has increased annually from 2008 to 2012, with 198 cases within outbreaks in 2012 alone [4].

Biofilm plays a significant role in infecting hosts and spreading bacteria [14,15]. It could be considered a strategy adopted by some bacteria to endure harsh conditions such as desiccation, host defense systems, and antibacterial agents [16,17]. *L. monocytogenes* can form biofilms on the surface of food machinery, food surfaces, and interiors, which can improve its resistance to external harmful substances and its adaptability to adverse environments, leading to food spoilage and endangering consumers’ health [18,19]. *L. monocytogenes* has brought a substantial economic burden and safety hazard to the food industry and public health [20,21]. In the early stage of biofilm formation, bacteria begin to produce flagella, which endow bacteria with the ability to adhere and move, making bacteria adhere to the surface of the host and promoting the formation of biofilm [22,23]. Extracellular polymer substances (EPSs) comprise extracellular DNA, proteins, and exopolysaccharides, which constitute more than 90% of biofilm components and endow bacteria with functions such as the ability to adhere to the host surface, form microcolonies and three-dimensional structures, and resist adverse external environments [24,25,26]. Proteins have been recognized as a major substance of biofilms, and they have essential functions in inducing inflammations and biofilm maintenance [27]. During the middle phase of biofilm formation, bacteria produce EPSs in large quantities to wrap themselves, which not only contribute to the establishment of biofilm but also promote growth by mediating cell attachment and expanding the volume of cell clusters [28,29,30]. Quorum sensing (QS) is a kind of intercellular communication mechanism between bacteria of the same or different species, mediated by autoinducers (AIs) produced by bacteria and diffusible small molecules that sense inducers [31,32,33]. At the stage of biofilm maturity, the phenomenon of quorum sensing begins to occur due to the interaction within the flora and develops into a complex spatial structure, showing stronger resistance [34,35]. At the final stage, the cells are separated from the aggregated biofilm to start another biofilm cycle [35,36]. The QS system of *L. monocytogenes* can regulate the expression of pathogenic virulence factors, biofilm formation, invasion, infection, and other behaviors [37,38].

Traditional prevention and control methods such as chemical disinfectants and fungicides cannot eradicate pathogenic bacteria and may also induce the generation of highly resistant bacteria [39,40]. At the same time, chemical preparations that are not wholly removed will also pose food safety hazards to consumers [41]. Therefore, the exploration and discovery of new and effective natural antibacterial substances present good application prospects [42,43]. Quorum sensing inhibitors (QSIs) show promise as a potential alternative to traditional antibiotics due to their high efficacy against drug-resistant strains and their ability to eliminate bacteria without promoting bacterial resistance [44,45].

Lactic acid bacteria (LAB) are recognized as safe probiotics, and their metabolites (organic acids, bacteriocins, hydrogen peroxide, carbon dioxide, etc.) can effectively inhibit pathogenic bacteria. [46,47]. LAB-derived quorum sensing inhibitors can inhibit the biofilm and QS of pathogenic bacteria and are considered potential substitutes for antibiotics [48]. For example, Aman et al. found that *Pediococcus pentosaceus* BS-2 and *Lactobacillus fermentum* BM-2 isolated from milk significantly inhibited the QS system and biofilm formation of *Pseudomonas aeruginosa* [49]. *Lacticaseibacillus rhamnosus* XN2, isolated from yak yoghurt, exhibited antibacterial activities against *Staphylococcus aureus*, *L. monocytogenes*, and *Escherichia coli*, and its bacteriocin was able to disrupt the cell membrane, inhibit the secretion of hemolysin, and regulate the QS system of *S. aureus* [50]. Melan et al. discovered that LAB extracts, acting as QSIs, were able to control multiple biological changes in *L. monocytogenes* and *S. aureus* to inhibit their infectivity and pathogenicity [51,52]. Extracts of *L. rhamnosus* showed potential as QSIs affecting *Vibrio parahaemolyticus* biofilm formation [53]. *Lactiplantibacillus plantarum* is one species of LAB with antibacterial properties [54]. de Lira et al. found that the cell-free supernatant of *L. plantarumm* was stable at different temperatures and pH values and effectively inhibited *L. monocytogenes* [55]. Although the inhibitory effect of *L. plantarumm* on *L. monocytogenes* has been confirmed, further research is needed to fully understand the inhibitory mechanisms at the transcriptome level.

In our previous study, 40 strains of LAB were isolated from Chaoshan tofu, fermented tempeh, milk tofu, pickled vegetables, and pickled beans, and their antibacterial activity against *L. monocytogenes* was measured, among which *L. plantarum* Z102 showed significant antibacterial and probiotic properties. In the present study, we explored the inhibitory effects of the purified active substance of *L. plantarum* Z102 (named as Z102-E) on *L. monocytogenes* growth, self-aggregation, surface hydrophobicity, sugar utilization, motility, QS, biofilm formation, and the contents of extracellular polysaccharides and extracellular proteins. Then, the transcriptomic analysis was conducted to reveal the action mechanism of Z102-E on *L. monocytogenes* biofilm at the molecular level, providing a theoretical basis and technical support for preventing and controlling *L. monocytogenes* contamination.

## 2. Materials and Methods

### 2.1. Preliminary Preparation

*L. plantarum* Z102 was cultured in De Man, Rogosa, and Sharpe (MRS) medium (Huankai, Guangzhou, China) at 37 °C without shaking for 12 h. *L. monocytogenes* ATCC 19115 and *Vibrio harveyi* BB170 were purchased from Guangdong Culture Collection Center of Microbiology (Guangzhou, China), and *L. monocytogenes* ATCC 19115 was incubated in Brain Heart Infusion (BHI) medium (Huankai) for 24 h at 37 °C (150 rpm), *V. harveyi* BB170 was incubated in Autoinducer Bioassay (AB) medium (Huankai) for 12 h at 30 °C (90 rpm).

*L. plantarum* Z102 was inoculated at 2% (*v*/*v*) in fresh MRS medium (2 L) and incubated at 37 °C for 18 h, and the bacterial suspension was centrifuged at 4 °C and 12,000 rpm for 30 min and filtered using 0.45 μm filters (Millipore; Boston, MA, USA). Then, the acellular supernatant was mixed with an equal amount of ethyl acetate overnight, the organic phase was collected, and the ethyl acetate was removed by rotary evaporation (R1001-VN; Great Wall Co., Ltd., Zhengzhou, China). The concentrated solution was freeze-dried (TGL-16gR; Anting technology Co., Ltd., Shanghai, China) and then subjected to Sephadex LH-20 gel chromatography (Solarbio technology Co., Ltd., Beijing, China) and semi-preparative high-performance liquid chromatography (PREP 150LC; Waters; Milford, MA, USA). The purity of the purified fraction was confirmed by high performance liquid chromatography (Aliance E2695; Waters). The high-purity fraction designated as Z102-E was obtained and subjected to LC-MS/MS (QTRAP 4500; AB SCIEX, Cupertino, CA, USA).

### 2.2. Analysis of Growth Curve and MIC

The suspension of *L. monocytogenes* ATCC 19115 was adjusted to 10^6^ CFU/mL, and Z102-E was dissolved in BHI medium and diluted to different concentrations (6.4, 3.2, 1.6, 0.8, 0.4, and 0.2 mg/mL). The media were sterilized through a 0.22 μm filter membrane (Millipore; Boston, MA, USA), and 200 μL of each medium was put in a 100-well plate. *L. monocytogenes* ATCC 19115 was inoculated at 2% into the plate wells and cultivated at 37 °C for 24 h. The OD at 600 nm was recorded by the automatic growth curve analyzer Bioscreen C (Labsystems; Helsinki, Finland). The minimum inhibitory concentration (MIC) of Z102-E referred to the lowest Z102-E concentration that completely inhibited the growth of *L. monocytogenes* ATCC 19115, which was determined according to reference [56].

### 2.3. Effects of Z102-E on Auto-Aggregation and Surface Hydrophobicity of L. monocytogenes

*L. monocytogenes* ATCC 19115 (10^6^ CFU/mL) was cultivated in BHI with 0, 8, 1.6, and 3.2 mg/mL of the Z102-E and subsequently centrifuged at 4 °C, 12,000 rpm. The auto-aggregation rate and surface hydrophobicity were analyzed according to the previously described assay [57,58].

### 2.4. The Effect of Z102-E on Carbohydrate Utilization of L. monocytogenes

Glucose, lactose, D-galactose, fructose, mannose, and maltose were used as the only carbohydrates (1%) in the tryptone soy broth (TSB) medium (5 mL) [59]. Z102-E was added in the sterilized media to the final concentrations of 6.4, 3.2, 1.6, 0.8, 0.4, and 0.2 mg/mL, respectively. The medium without Z102-E was used as a control. *L. monocytogenes* ATCC 19115 was inoculated at 2% and incubated at 37 °C for 24 h. Then, the OD_600nm_ was determined.

### 2.5. Motility Assay

The *L. monocytogenes* ATCC 19115 suspension (10^6^ CFU/mL) was dipped and pierced vertically in BHI semi-solid medium (0.4% agar) with different Z102-E concentrations (3.2, 1.6, 0.8, and 0 mg/mL) and cultured at 25 °C for 48 h. The motility of *L. monocytogenes* was observed [60].

### 2.6. Effect of Z102-E on AI-2 Signal Molecules of L. monocytogenes

*V. harveyi* BB170 is a standard luminescent strain that emits light in the presence of AI-2 signal molecules [61]. In this study, the AI-2 signal molecule was detected by the bioluminescence method of *V. harveyi* BB170 according to a previous study [61]. The *L. monocytogenes* ATCC 19115 suspension (10^6^ CFU/mL) was inoculated at 2% in BHI with 0, 8, 1.6, and 3.2 mg/mL of Z102-E and incubated at 37 °C for 24 h, then centrifuged at 4 °C, 12,000 rpm for 10 min and filtered using 0.45 μm filters (Millipore; Boston, MA, USA), and the cell-free supernatant was obtained. The *V. harveyi* BB170 suspension was diluted by fresh AB medium at 1:5000; then, the cell-free supernatant of *L. monocytogenes* ATCC 19115 was mixed with *V. harveyi* BB170 suspension at a 1:50 ratio.

After reacting for 3 h at 100 rpm at 30 °C, 200 μL of the mixture was put into a 96-well black enzyme plate. The acellular supernatant of *V. harveyi* BB170 was used as a positive control, aseptic AB medium was used as a negative control, and aseptic BHI was used as a medium control. The continuous-wavelength multi-function microplate detection platform (SpectraMax i3x; Molecular Devices, Sunnyvale, CA, USA) was used to determine the bioluminescence of *V. harveyi* BB170.

### 2.7. Effect of Z102-E on Biofilm Formation of L. monocytogenes

Biofilm formation was assessed using the crystal violet (CV) staining assay, as described by Govaert [62]. *L. monocytogenes* ATCC 19115 aliquots (10^6^ CFU/mL) were cultured in 200 μL BHI supplemented with varying concentrations of Z102-E (0, 0.8, 1.6, and 3.2 mg/mL). Following 24 h of static incubation at 37 °C, the plate wells were rinsed thrice with phosphate-buffered saline (PBS, pH 7.2), air-dried at 60 °C for 30 min, and then stained with 0.1% (*w*/*v*) crystal violet for 5 min at room temperature. Subsequently, the plate wells were rinsed with PBS (pH 7.2), and glacial acetic acid (Macklin, Shanghai, China) was added to dissolve the stain. The absorbance was measured at 595 nm.

### 2.8. Removal Effects of Z102-E on the Mature Biofilm of L. monocytogenes

*L. monocytogenes* ATCC 19115 (10^6^ CFU/mL) was inoculated into fresh aseptic BHI medium and cultured at 37 °C for 48 h to develop a mature biofilm. Then, the upper suspension was discarded, and the planktonic cells on the biofilm surface were washed off by PBS. The 200 μL of Z102-E dilutions in BHI at concentrations of 25.6, 12.8, 6.4, and 3.2 mg/mL were added to the plate wells and incubated at 37 °C for 4, 24, and 48 h. The absorbance was measured at 595 nm [53].

### 2.9. Quantification of Extracellular Polysaccharides in Biofilm

Different concentrations of 1 mL of Z102-E dilutions in BHI (3.2, 1.6, 0.8, and 0 mg/mL) were put into a 24-well plate. *L. monocytogenes* ATCC 19115 (10^6^ CFU/mL) were inoculated into the well and added with sterile coverslips (12 mm × 12 mm). After incubation for 24 h at 37 °C, the biofilms on the coverslips were rinsed with 1 mL of 0.9% NaCl for 30 s, and 1 mL of the rinsed solution and 1 mL of 5% phenol were mixed and then added to 5 mL of 98% H_2_SO_4_ and incubated for 10 min. Then, the absorbance at 490 nm was determined [63].

### 2.10. Effect of Z102-E on Extracellular Proteins in L. monocytogenes Biofilm

The Bradford Protein Assay Kit (Solebao Co., Ltd., Beijing, China) was applied to measure the extracellular protein content of *L. monocytogenes* ATCC 19115 following the manufacturer’s protocol. *L. monocytogenes* ATCC 19115 was inoculated in 1 mL BHI medium with different Z102-E concentrations (12.8, 6.4, 3.2, 1.6, 0.8, 0.4, and 0.2 mg/mL) at the inoculation ratio of 2% and cultured at 37 °C for 24 h to form biofilm. Standard solutions with different concentrations were prepared, their OD was determined at 595 nm, and the standard curve was plotted. From the standard curve, the protein concentrations of the biofilm samples were calculated [64].

### 2.11. Observation of Biofilm by Inverted Fluorescence Microscopy

An amount of 12 μL of 10^6^ CFU/mL of *L. monocytogenes* ATCC 19115 was added in 600 μL of BHI with 0, 0.8, 1.6, and 3.2 mg/mL of Z102-E and cultured in 8-compartment cell culture slides at 37 °C for 24 h. After gentle washing with PBS, the biofilms were stained using the Live/Dead Cell Viability Assay Kit (Uelandy technology, Co., Ltd., Suzhou, China) and concanavalin (Thermo Fisher Scientific, Waltham, MA, USA), respectively, and observed with an inverted fluorescence microscope (Axio Observer A1; Zeiss, Oberkochen, Baden-Wurttemberg, Germany) after 15 min of treatment in the dark.

### 2.12. Transcriptomic Analysis

Three replicates of *L. monocytogenes* ATCC 19115 biofilm samples were collected from the control group (C) and the Z102-E (0.8 mg/mL)-treated group (T) and then preserved at −80 °C, respectively, for transcriptomic analysis. Total RNA was extracted from *L. monocytogenes* ATCC 19115 samples using the Tissue Total RNA Kit (Yeasen, Shanghai, China). The purity and concentration of the RNA samples were determined using Nanodrop 2000 (Thermo Fisher Scientific), and the mRNA was fragmented to construct a library by using the TruSeqTM Total RNA Library Prep Kit (Illumina, San Diego, CA, USA) and sequenced by Illumina Hiseq (NovaSeqXPlus; Illumina, San Diego, CA, USA); then, the cleaned reads were obtained by Bowtie 2 (http://bowtie-bio.sourceforge.net/index.shtml (accessed on 24 December 2022). DESeq2 (http://bioconductor.org/packages/stats/bioc/DESeq2 (accessed on 24 December 2022) was used to analyze the differential expressions of genes between samples, using Padjust < 0.05 and |log_2_FC| > 1 as conditions to screen out differentially expressed genes (DEGs). Using Transcripts Per Million reads (TPM) as an indicator, the software RSEM v1.3.3 (http://bioconductor.org/packages/stats/bioc/DESeq2 (accessed on 25 December 2022) was used to analyze DEGs. The function annotation and pathway enrichment analysis were performed using Gene Ontology (GO) (http://www.geneontology.org/ (accessed on 25 December 2022) and Kyoto Encyclopedia of Genes and Genomes (KEGG) (http://www.genome.jp/kegg/ (accessed on 25 December 2022).

### 2.13. Data Analysis

Data analysis and graph drawing were performed using SPSS 25.0 (IBM, Armonk, NY, USA) and GraphPad prism 8.0 (GraphPad Software, San Diego, CA, USA). In all the figures, data are shown as mean ± SD (*n* = 3), and error bars represent standard errors.

## 3. Results

### 3.1. The Antibacterial Effect of Z102-E against L. monocytogenes

**Figure 1:**
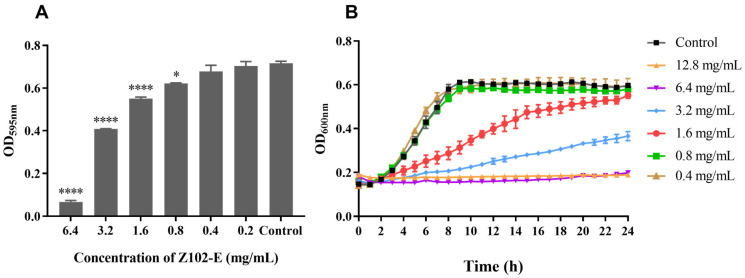
The antibacterial efficacy of Z102-E against *L. monocytogenes*. (**A**) The effects of Z102-E on *L. monocytogenes* growth. (**B**) The growth curves of *L. monocytogenes* treated with different concentrations of Z102-E. The significance levels are shown as * *p* < 0.05 and **** *p* < 0.0001 compared to the data of control.

The purity analysis and LC-MS/MS identification of Z102-E are shown in Figures S1 and S2. The single peak was observed in the high-performance liquid chromatography, indicating that Z102-E was pure. LC-MS/MS analysis indicated that Z102-E is a peptide with the amino acid composition of TGVWEVK. Figure 1 indicates that the MIC of Z102-E against *L. monocytogenes* was 6.4 mg/mL. In order to investigate the anti-biofilm effects of Z102-E, the sub-MICs (0.8, 1.6, and 3.2 mg/mL) were chosen for the subsequent anti-biofilm experiments.

### 3.2. Effects of Z102-E on the Self-Aggregation and Surface Hydrophobicity of L. monocytogenes

**Figure 2:**
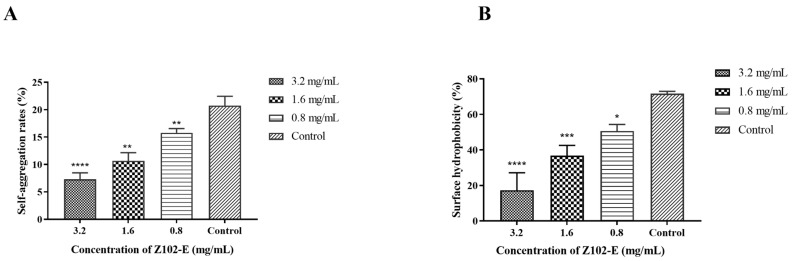
The effects of Z102-E on the self-aggregation and surface hydrophobicity. (**A**) Inhibition effects on self-aggregation. (**B**) Inhibition effects on surface hydrophobicity. The significance levels are shown as * *p* < 0.05, ** *p* < 0.01, *** *p* < 0.001, and **** *p* < 0.0001 compared to the data of control.

Self-aggregation is one of the virulence factors of foodborne pathogens; it plays a role in the bacterial invasion of intestinal epithelial cells and positively correlates with bacterial adhesion [57]. After treatment with different concentrations of Z102-E, the self-aggregation and surface hydrophobicity of *L. monocytogenes* were significantly reduced (Figure 2). When treated with 3.2 mg/mL, 1.6 mg/mL, and 0.8 mg/mL of Z102-E, the bacterial self-aggregation rates were 7.31%, 10.66%, and 15.77%, respectively, which were significantly lower than that of the control (*p* < 0.05) (Figure 2A). The surface hydrophobicity of foodborne pathogens is a hallmark of the virulence of foodborne pathogens and one of the important driving forces that determine the nonspecific adhesion of bacteria at various biotic and abiotic interfaces [36]. Under 3.2, 1.6, and 0.8 mg/mL of Z102-E, the surface hydrophobicities were 17.29, 36.80, and 50.63%, respectively (Figure 2B). This shows that the self-aggregation and surface hydrophobicity of *L. monocytogenes* were significantly reduced after Z102-E treatment and that there was a dose–response relationship.

### 3.3. The Influence of Z102-E on Carbohydrate Utilization of L. monocytogenes

**Figure 3:**
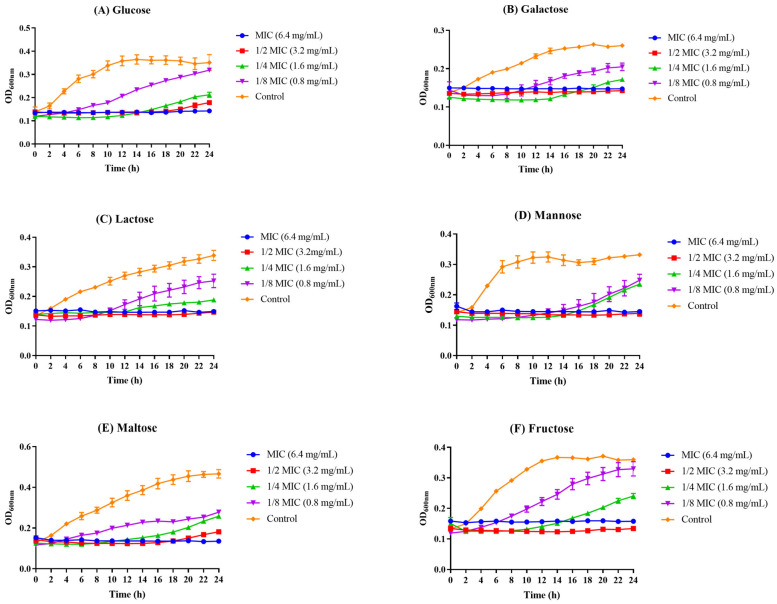
The effects of Z102-E on the utilization of different sugars of *L. monocytogenes*. (**A**–**F**) refer to different sugars.

In TSB medium, six sugars were selected as the only carbohydrates, and Z102-E was found to have the ability to inhibit the utilization of these sugars by *L. monocytogenes*. Under the action of a low concentration of Z102-E, *L. monocytogenes* can utilize sugars for metabolism and energy conversion. With the increase in Z102-E concentration, the absorption and utilization of these six sugars by *L. monocytogenes* were limited, which indicated that Z102-E could inhibit the utilizations of different carbohydrates by *L. monocytogenes* (Figure 3).

### 3.4. The Inhibition on Motility

**Figure 4:**
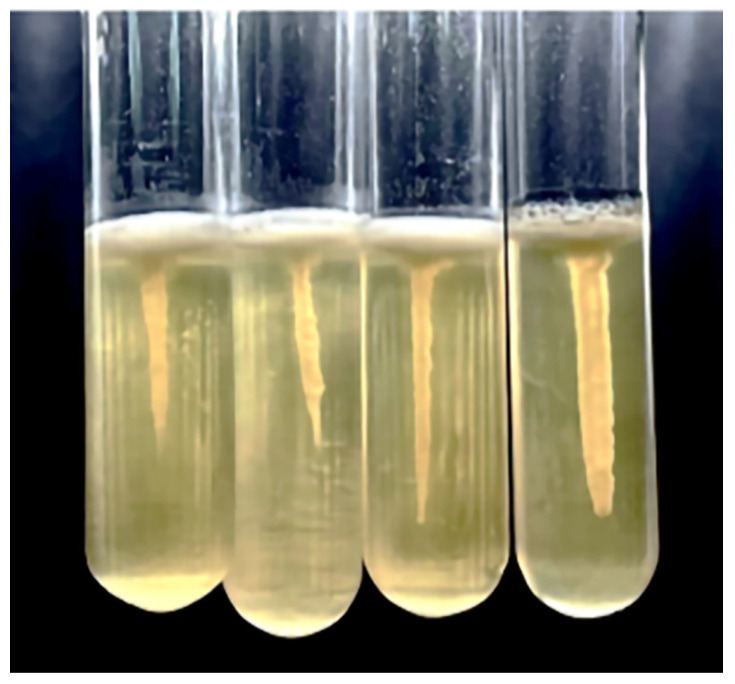
The Z102-E effect on motility of *L. monocytogenes*. The concentrations of Z102-E from left to right are 3.2, 1.6, 0.8, and 0 mg/mL.

Motility significantly affects biofilm formation, and it is also a critical factor in bacterial pathogenicity [60]. In this study, the influence of Z102-E on the motility of *L. monocytogenes* was determined by semi-solid stab culture experiments. After Z102-E treatment, *L. monocytogenes* formed smaller umbrella-shaped growth lines than the control (Figure 4).

### 3.5. Influence of Z102-E on Biofilm Formation of L. monocytogenes

**Figure 5:**
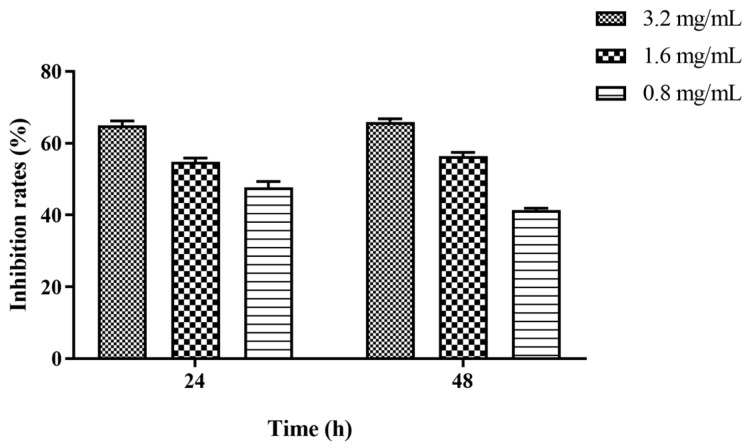
The effects of Z102-E at sub-MICs on the biofilm of *L. monocytogenes*.

Different concentrations of Z102-E were found to remarkably suppress the formation of *L. monocytogenes* biofilm and reduce the biofilm biomass, demonstrating a dose–response relationship. After 24 h treatment with 3.2 mg/mL and 1.6 mg/mL Z102-E, the amount of biofilm decreased by 64.97% and 54.78%, respectively (Figure 5). When the treatment time reached 48 h, the inhibition rates of the biofilm were 65.97% and 56.38%.

### 3.6. Elimination Effects of Z102-E on the Mature Biofilm of L. monocytogenes

**Figure 6:**
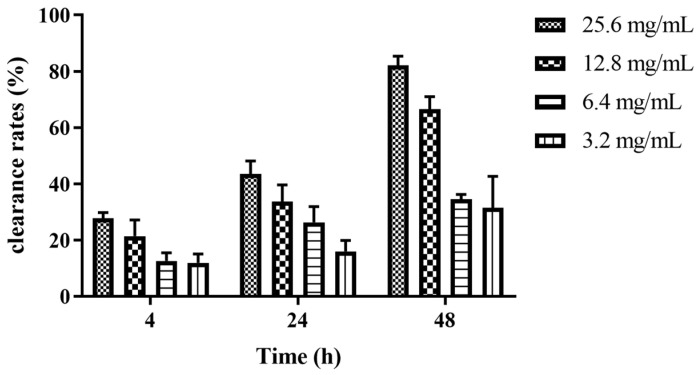
The removal effects of Z102-E on the mature biofilm of *L. monocytogenes*.

Different concentrations of Z102-E exhibited obvious effects on the removal of the *L. monocytogenes* biofilm, and the removal rate was positively correlated with the concentration of Z102-E and the treatment time. When treated at 25.6 mg/mL and 12.8 mg/mL, the removal rates were 20.85% and 21.43% at 4 h, 30.08% and 33.76% at 24 h, and 62.07% and 66.62% at 48 h, respectively. Z102-E showed an excellent removal effect at 48 h (Figure 6).

### 3.7. Effect of Z102-E on AI-2 Signal Molecule, Extracellular Proteins, and Extracellular Polysaccharides of L. monocytogenes Biofilm

**Figure 7:**
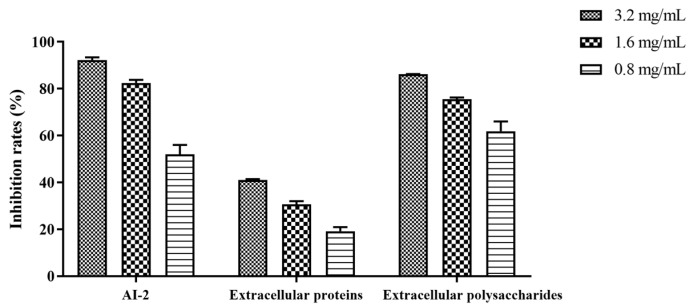
The effects of Z102-E on AI-2 signal molecule, extracellular proteins, and extracellular polysaccharides of *L. monocytogenes* biofilm.

The AI-2 system is a quorum-sensing signal shared by Gram-negative and Gram-positive bacteria; it is critical for regulating bacterial motility and biofilm formation [31,32]. The AI-2 signal molecules were inhibited by Z102-E at 3.2 mg/mL and 1.6 mg/mL of Z102-E, and the inhibition rates were 60.18% and 82.44%, respectively (Figure 7).

Different concentrations of Z102-E were measured for their abilities to inhibit the extracellular protein production of *L. monocytogenes*. Treatment with 3.2 mg/mL and 1.6 mg/mL of Z102-E showed inhibition rates of 41.07% and 19.16%, respectively (Figure 7). These results suggested that Z102-E presented a notable inhibitory effect on the extracellular protein production of *L. monocytogenes*.

Extracellular polysaccharide plays a critical role in preserving the intricate spatial structure of biofilms, inhibiting the diffusion of antibacterial agents into the biofilm, and enhancing biofilm resistance to drugs [24,25]. The content of *L. monocytogenes* extracellular polysaccharide decreased gradually with the increase in inhibitor concentration. When the biofilms were treated with 3.2 mg/mL and 1.6 mg/mL of Z102-E, the inhibition rates on the extracellular polysaccharides were 75.55% and 67.51%, respectively (Figure 7). The results also confirmed the inhibitory influence of Z102-E on *L. monocytogenes* biofilm formation.

### 3.8. Inverted Fluorescence Microscopy Observation of the Cells and EPS in Biofilm

**Figure 8:**
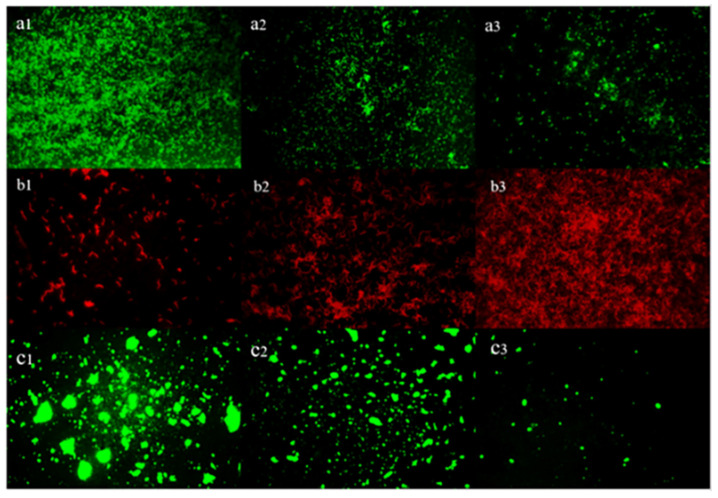
Inverted fluorescence microscopy observation of cells and EPS in biofilm (400×). (**a**) live cells; (**b**) dead cells; (**c**) EPS; subscripts 1–3 indicate different concentrations of Z102-E (0, 1.6, and 3.2 mg/mL).

The biofilm treated with Z102-E was visualized using the live/dead bacteria staining kit and concanavalin. The biofilm in the control group exhibited good activity and high EPS production, with bacterial cells encircled by a large amount of EPS. The biofilm structure was complete, dense, and thick. Under increasing concentrations of Z102-E, the survival rate of the cell and EPS amount decreased obviously, and the number of dead cells increased. Meanwhile, the bacterial growth was limited, appearing in small fragments (Figure 8). We can speculate that Z102-E could effectively inhibit *L. monocytogenes* biofilm, cause cell damage, and destroy EPS.

### 3.9. Analysis of DEGs

Transcriptomics was used to analyze the gene expression differences between the Z102-E-treated group and the control. The quality control results indicated that the sequencing data of all the samples could be further used for bioinformatics analysis (Tables S1 and S2). A total of 2931 internal reference genes were identified, with 331 showing significantly differential expression (|log_2_FC| ≥ 1.0 and *p*-adjusted < 0.05). Among them, 117 genes (3.99%) were up-regulated, while 214 genes (7.30%) were down-regulated.

**Figure 9:**
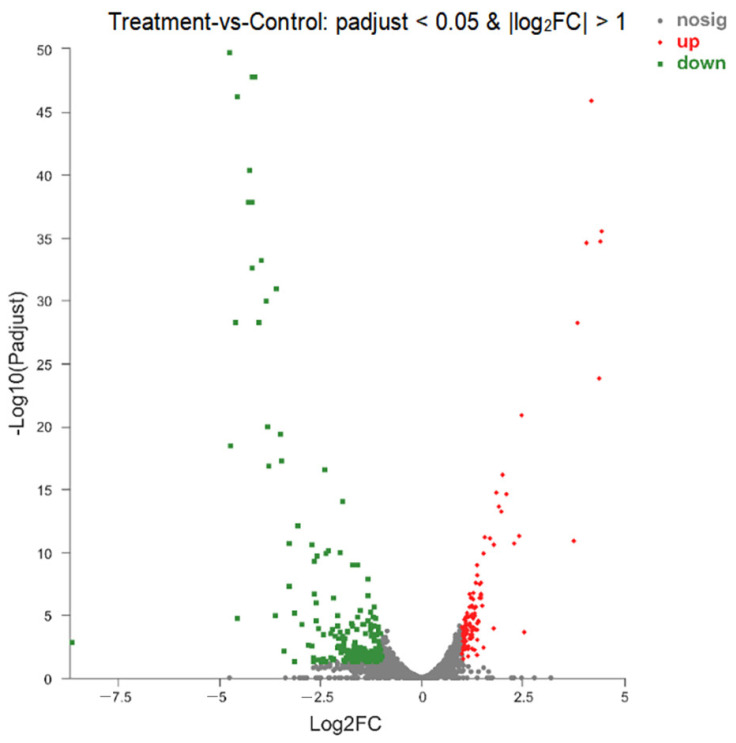
Volcano plot of DEGs. Green—significantly down-regulated genes, red—significantly up-regulated genes, gray—genes with no significant changes.

Notable DEGs are presented in Table 1 and Table 2, representing the top 8% of the up- and down-regulated genes. The higher number of down-regulated genes compared to up-regulated genes could be attributed to the suppressive impact of Z102-E on *L. monocytogenes* transcription (Figure 9).

The results showed that Z102-E regulated nine enzyme-related genes, including genes related to short-chain dehydrogenase, sugar-phosphate isomerase, transaldolase, transketolase, dihydroxyacetone kinase, triosephosphate isomerase, diol dehydratase subunit, cob(I) alamin adenosyltransferase PduO, and propanediol dehydratase subunit alpha. Among the significantly down-regulated genes, 43 genes were related to metabolic pathways, especially to the organic acid metabolic process and organic carboxyl compound metabolic process, and 22 genes often encoded cell membrane proteins. In addition, the expressions of several functional proteins were regulated by Z102-E, such as glycine cleavage system aminomethyl transferase T, flagellar biosynthetic protein, flagellar basal body rod protein, amino acid ABC transporter ATP-binding protein, phosphotransferase system (PTS) mannose transporter subunit IIA, ABC transporter ATP-binding protein, thiamin biosynthesis protein, PduA protein, and PduB protein, which implied that the treatment significantly affected the normal metabolism of *L. monocytogenes.*

### 3.10. Cluster Analysis

**Figure 10:**
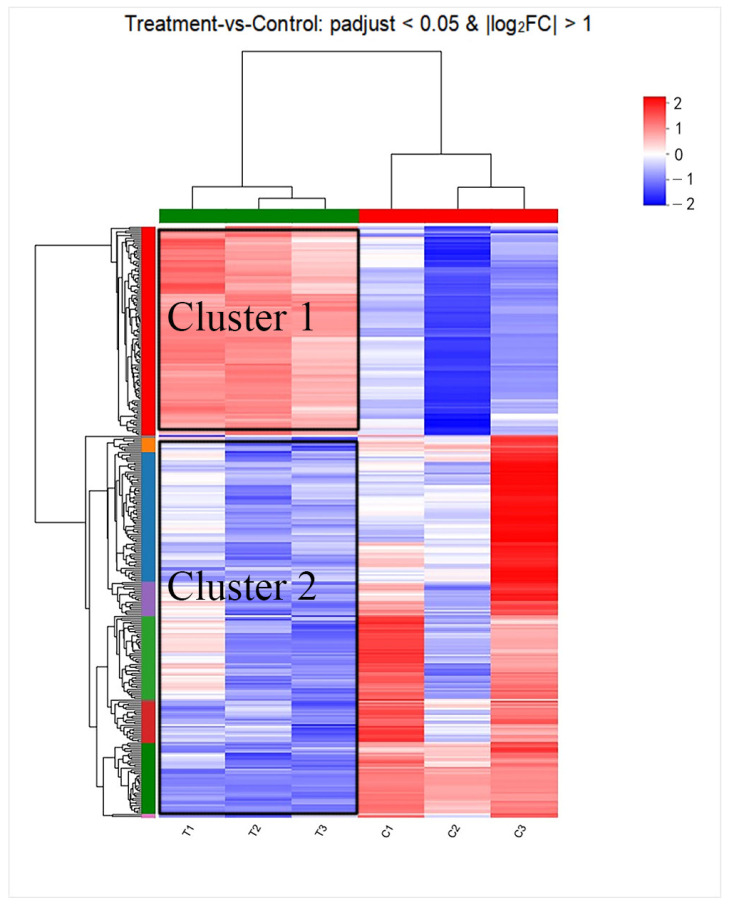
Heatmap of DEGs. Different rows and columns represent different genes and groups of samples, respectively.

Distance matrices were generated to represent the distance between samples. Genes sharing similar biological functions often clustered together. To visually analyze the significant DEGs between the control group and the Z102-E-treated group, we performed a cluster analysis on these DEGs. As shown in Figure 10, unsupervised clustering resulted in the formation of two main gene clusters. In the Z102-E-treated group, Cluster 1 comprised 35.35% of the DEGs, with 117 up-regulated genes, while Cluster 2 accounted for 64.65% of the DEGs and contained 214 down-regulated genes.

### 3.11. Gene Ontology (GO) Functional Analysis

The GO annotations indicated that DEGs in the *L. monocytogenes* biofilm encompassed three main functional categories: biological process (BP), cellular component (CC), and molecular function (MF). In our study, 40 secondary GO terms were identified, with 9 related to BP, 9 to CC, and 22 to MF (Figure S3), with 331 DEGs annotated, revealing that these genes may be associated with multiple GO terms (Table S3). The DEGs were mainly concentrated in the membrane integral component (25.38%), plasma membrane (16.31%), cytoplasm (11.78%), ATP binding (10.27%), and metal ion binding (7.25%), and most of the genes involved in these functional categories were down-regulated (Figure S4). In addition, the significantly down-regulated genes were also concentrated in DNA binding, the phosphoenolpyruvate-dependent sugar phosphotransferase system, the carbohydrate metabolic process, etc. (Figure S4).

### 3.12. GO Enrichment Analysis

**Figure 11:**
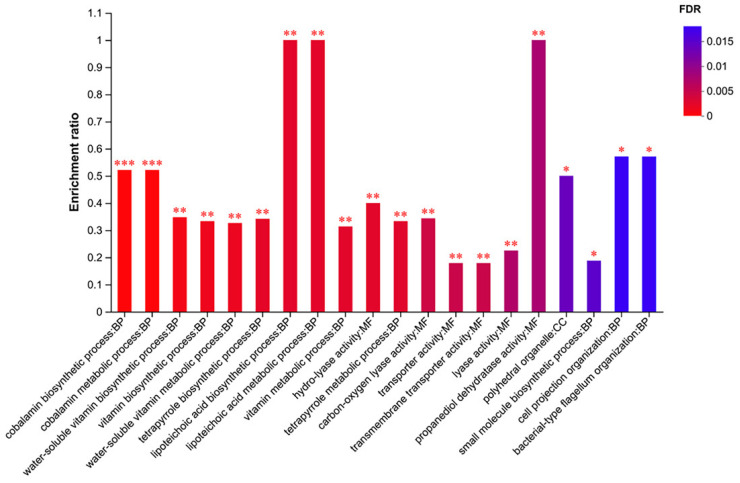
The top 20 GO enrichment terms. Enrichment ratio is the ratio of the number of genes annotated to the specific pathway (sample number) to all the genes annotated to the pathways (background number). FDR is a calibrated *p*-value indicating significance. *** FDR < 0.001, ** FDR < 0.01, * FDR < 0.05.

The analysis of the GO functional enrichment of the DEGs demonstrated the distinctions in gene function between the samples. The top 20 functional enrichments shown in Figure S5 and Figure 11 were predominantly involved in BP, specifically in the lipoteichoic acid biosynthetic process, lipoteichoic acid metabolic process, cobalamin biosynthetic process, cobalamin metabolic process, and hydro-lyase activity.

### 3.13. KEGG Function Analysis

KEGG analysis reveals the profile of genes associated with each specific metabolic pathway, which is helpful in revealing the anti-biofilm mechanism of Z102-E against *L. monocytogenes*. As shown in Figure S6, a total of 136 DEGs were involved in the biochemical metabolism or signal transduction pathways of *L. monocytogenes*. In the KEGG pathway, most of the DEGs were involved in carbohydrate metabolism, metabolism of cofactors and vitamins, membrane transport, lipid metabolism, cell motility, and antimicrobial drug resistance (*dlt*A, *dlt*B, *dlt*C, *dlt*D, and *ami*ABC), indicating that Z102-E could notably affect the metabolism, motility, and drug resistance of *L. monocytogenes*, thereby causing cell damage.

### 3.14. KEGG Enrichment Analysis

The analysis of the KEGG pathway enrichment revealed that out of the top 12 pathways, 14 DEGs were associated with the PTS (*bgl*F and *cel*B) and ABC transporters (*mod*B, *lpl*B, *bmp*A, *tcy*L, *tcy*N, *mnt*B, *mnt*A, *znu*B, *cbi*O, and *mdl*A), showing the highest levels of enrichment, and porphyrin and chlorophyll metabolism (*cbi*K, *cob*J, *cob*M, *cb*iG, *cb*iD, *cb*iB, and *cob*D) and starch and sucrose metabolism were the second most enriched (Figure S7). Propanoate metabolism, fructose and mannose metabolism (*fru*A, *fba*A, *gut*B, and *rha*B), and the two-component system (*pdta*R, *uhp*T, and *che*A) were also significantly enriched. The cationic antimicrobial peptide (CAMP) resistance, valine, leucine, and isoleucine biosynthesis (*leu*B, *ilv*C, *ilv*D, and *leu*C), flagellar assembly (*fli*l, *fli*P, *flh*A, *flh*B, and *flg*G), gluconeogenesis, pentose and glucuronic acid interconversion (*gu*tB, *rpe,* and *rha*B), and other related pathways were enriched by the remaining DEGs. The biofilm formation of *L. monocytogenes* and the QS regulation were related to the two-component system, glucose metabolism, cell secretion system, phosphotransferase system, flagella assembly, and changes in the carbohydrate metabolism pathways. We speculated that Z102-E was effective in inhibiting biofilm formation and regulating QS-related metabolic pathways.

## 4. Discussion

The results revealed that Z102-E effectively reduced *L. monocytogenes* self-aggregation, surface hydrophobicity, sugar utilization, motility, biofilm formation, AI-2 signal molecules, and the contents of extracellular polysaccharides and extracellular protein. The analysis of the DEGs in the *L. monocytogenes* biofilm treated with sub-MIC of Z102-E showed significant expression changes in 331 genes, with 117 up-regulated and 214 down-regulated. These DEGs were found to be mainly involved in the PTS system, two-component system, motility, drug resistance, amino acid metabolism, and vitamin metabolism. These findings provide valuable insights into the molecular mechanisms related to the response of the pathogen to sub-MIC levels of Z102-E, contributing to our understanding of biofilm formation and potential strategies for combating *L. monocytogenes* contamination.

For carbohydrate metabolism, the main down-regulated pathways in the treatment group were pentose and glucuronic acid interconversion (*gu*tB, *rpe*, and *rha*B) and fructose and mannose metabolism (*fru*A, *fba*A, *gut*B, and *rha*B). The PTS is ubiquitously found in bacteria and is mainly responsible for the transport and phosphorylation of sugars for bacterial energy [65]. It is also the primary system for the uptake of sugars by bacteria [66]. Many of the intermediates of PTS have been associated with various regulatory factors such as the virulence factors [67]. Analysis of the annotated DEGs revealed significant down-regulations of *bgl*F and *cel*B, which encoded beta-glucosidaseF and exoglucanase, respectively [68,69]. It indicates that Z102-E can significantly reduce the expression level of some PTS membrane transport genes related to saccharides and inhibit the bacterial uptake of carbon sources, thus affecting the material metabolism and energy conversion of the cell, which is in line with the influence of Z102-E on the carbon source utilization of *L. monocytogenes*.

The motility of bacterial flagella is crucial in the initial phase of bacterial biofilm formation, particularly during the adhesion period, and it significantly aids pathogenic bacteria in invasion [22,23]. Additionally, the flagellar movement not only helps in the invasion process but also plays a vital role in enabling bacteria to detach from mature biofilms [35,36]. Flagellar synthesis is regulated by many genes and is a more complex process [70]. Deletion of the *fli*P gene leads to a loss of flagellar structure, affecting bacterial motility [71]. In this study, the genes *fli*l, *fli*P, *flh*A, *flh*B, and *flg*G related to flagella assembly and *che*A related to bacterial chemotaxis were significantly down-regulated after *L. monocytogenes* was treated with Z102-E, which was consistent with the mobility experiment results.

The EPS of biofilm plays crucial roles in surface adhesion initiation, cell cluster formation, and in stabilizing the complex multi-layer biofilm structure [72]. In this study, 22 significantly down-regulated genes often encoded cell membrane proteins. The ABC transport system is a family of proteins that transfers substances in and out of cells through the lipid bilayer of the cell membrane by consuming ATP for energy [73]. It directly or indirectly gets involved in the process of biological periplasm formation and exerts influence on bacterial substance transfer [74,75]. We found that the DEGs related to the ABC transport system that were significantly down-regulated by Z102-E treatment mainly include *mod*B, *lpl*B, *bmp*A, *tcy*L, *tcy*N, *mnt*B, *mnt*A, *znu*B, *cbi*O, and *mdl*A.

The two-component system is ubiquitous in bacteria to control gene expressions through autophosphorylation cascades to regulate cellular signal transduction [76]. It regulates bacterial QS, biofilm formation, drug resistance, and virulence and promotes bacterial activity and invasion [77]. In this study, the genes *pdta*R, *uhp*T, and *che*A associated with the two-component system were significantly down-regulated. We speculate that Z102-E can regulate and control the QS of *L. monocytogenes* and enhance bacterial stress resistance by controlling the expressions of the genes involved in the two-component system.

Bacterial stress resistance causes huge economic losses and human deaths every year. Many bacteria have been found to be highly resistant to fluoroquinolones, tetracyclines, and β-lactams [78]. The two-component system endows bacteria with a rapid response to antibiotics and enhances bacterial resistance [79]. VanS is a sensor histidine kinase that detects vancomycin and then activates VanR, which in turn directs the expression of vancomycin resistance genes [80]. In this study, we found that *dlt*A, *dlt*B, *dlt*C, and *dlt*D associated with the two-component system of *L. monocytogenes* were down-regulated after treatment with Z102-E, and the gene *ami*ABC related to antibiotic resistance was significantly down-regulated. It indicated that Z102-E can improve the sensitivity of *L. monocytogenes* to antibiotics and reduce its antibiotic resistance.

The metabolisms of amino acids, cofactors, and vitamins are the fundamental characteristics of bacteria [81,82,83,84]. For the amino acid metabolism, the pathways down-regulated by the treatment with Z102-E mainly include valine, leucine, and isoleucine biosynthesis (*leu*B, *ilv*C, *ilv*D, and *leu*C). For the metabolism of the cofactors and vitamins, the main down-regulated pathways in the treatment group were porphyrin and chlorophyll metabolism (*cbi*K, *cob*J, *cob*M, *cb*iG, *cb*iD, *cb*iB, and *cob*D). It was found that Z102-E can regulate the metabolisms of *L. monocytogenes*, thereby affecting the energy supply and inhibiting *L. monocytogenes* growth.

## 5. Conclusions

In summary, the *L. plantarum* extract Z102-E exerted efficient anti-biofilm effects against *L. monocytogenes*. The sub-MIC treatment of Z102-E resulted in the decreases of motility, AI-2 signal production, EPS production, and biofilm formation in *L. monocytogenes*. The anti-biofilm mechanism of Z102-E against *L. monocytogenes* was revealed by transcriptome analysis; the RNA-seq results confirmed that Z102-E could impede biofilm formation by suppressing the expressions of quorum sensing genes, and other biofilm-associated genes. These findings present a promising strategy for reducing stress resistance and the persistent contamination of *L. monocytogenes* biofilm. However, the DEGs identified by transcriptomics should be further validated, and it is important to deeply investigate the specific mechanisms by which Z102-E impacts *L. monocytogenes* biofilm. In the future, we will conduct further study on the wild-type strain, deletion mutant, and complementary strain of *L. monocytogenes* and combine proteomic and metabolomic methods to further analyze how Z102-E effectively controls the biofilm of *L. monocytogenes*.

## Figures and Tables

**Table 1:** Top significantly up-regulated genes.

Gene Name	Gene Description	FC (T/C)	Log_2_FC (T/C)
*lmo0344*	short chain dehydrogenase	22.077	4.464464
*lmo0345*	sugar-phosphate isomerase	21.196	4.405722
*lmo0343*	transaldolase	21.032	4.394532
*lmo0342*	transketolase	18.269	4.191303
*lmo0347*	dihydroxyacetone kinase	16.727	4.064068
*lmo0348*	dihydroxyacetone kinase	14.344	3.842421
*lmo1249*	hypothetical protein	13.607	3.766271
*lmo1997*	PTS mannose transporter subunit IIA	5.734	2.51942
*lmo0346*	triosephosphate isomerase	5.534	2.468207

“C” is the control group; “T” is the treated group.

**Table 2:** Top significantly down-regulated genes.

Gene Name	Gene Description	FC (T/C)	Log_2_FC (T/C)
*-*	hypothetical protein AVP43_02707 [*Geobacillus stearothermophilus*]	0.002	−8.673013
*lmo1190*	hypothetical protein	0.036	−4.779108
*lmo1151*	PduA protein	0.038	−4.724699
*lmo1152*	PduB protein	0.041	−4.602022
*lmo1162*	hypothetical protein	0.042	−4.573928
*lmo1154*	diol dehydratase subunit gamma	0.042	−4.571224
*lmo1164*	ATP:cob(I)alamin adenosyltransferase PduO	0.051	−4.299481
*lmo1153*	propanediol dehydratase subunit alpha	0.052	−4.266549
*lmo1158*	PduK protein	0.054	−4.211739
*lmo1156*	diol dehydratase-reactivating factor large subunit	0.054	−4.200381
*lmo1165*	ethanolamine utilization protein EutE	0.055	−4.189399
*-*	hypothetical protein LM700514_40645 [*Listeria monocytogenes*]	0.057	−4.133942
*lmo1161*	ethanolamine utilization protein EutJ	0.06	−4.054854
*lmo1166*	NADPH-dependent butanol dehydrogenase	0.064	−3.963328
*lmo1155*	diol dehydratase subunit gamma	0.07	−3.842152
*lmo1159*	carboxysome structural protein	0.07	−3.827296
*lmo1160*	PduL protein	0.073	−3.778299
*lmo1163*	carbon dioxide concentrating mechanism protein	0.081	−3.623202

“C” is the control group; “T” is the treated group.

## Data Availability

The original contributions presented in the study are included in the article/Supplementary Material, further inquiries can be directed to the corresponding author.

## Supplementary Materials

The following supporting information can be downloaded at: https://www.mdpi.com/article/10.3390/foods13162495/s1, Table S1: The results of quality control of RNA-Seq data, Table S2: Comparison of samples with the genome of *L. monocytogenes* EGD-e, Table S3: GO annotation results of *L. monocytogenes* DEGs, Figure S1: HPLC purification chromatogram of Z102-E, Figure S2: LC-MS/MS analysis of Z102-E, Figure S3: GO categorization, Figure S4: Classification of up-regulated and down-regulated genes in GO, Figure S5: GO enrichment analysis, Figure S6: KEGG pathway classification, Figure S7: KEGG enrichment analysis.
