# Supplementary material for: Anti-Biofilm Effects of Z102-E of Lactiplantibacillus plantarum against Listeria monocytogenes and the Mechanism Revealed by Transcriptomic Analysis
Source: Foods. 2024 Aug 8;13(16):2495. doi: 10.3390/foods13162495 (PMC11354177; doi:10.3390/foods13162495)
Supplement: Supplementary file 1 [file foods-13-02495-s001.zip › foods-3113166-supplementary.pdf]

Table S1. The results of quality control of RNA-Seq data

Sample Name	Raw reads	Clean Reads	Clean Bases (bp)	Clean Error Rate (%)	Clean Q20(%)	Clean Q30(%)
C1	30474390	29769124	4150903261	0.0255	97.88	93.52
C2	26685280	26352816	3828751947	0.0257	97.84	93.38
C3	29427118	28772746	3940570076	0.0251	98.03	93.93
T1	23159812	22991940	3342980647	0.0261	97.68	92.98
T2	23503720	23065238	3223483903	0.0255	97.95	93.58
T3	26874534	26663626	3711065588	0.0256	97.86	93.49

Q20 and Q30, respectively, represented the percentage of bases with Phred values greater than 20 and 30 in the total bases after data quality control. C: control group; T: treated group with 0.8 mg/mL Z102-E.

Table S2. Comparison of samples with the genome of *Listeria monocytogenes* EGD-e

Sample Name	Total Reads	Genome Mapped Ratio (%)	Unmapped Reads Ratio (%)	Uniq Mapped Reads Ratio (%)
C1	29769124	82.59	17.41	67.26
C2	26352816	93.55	6.45	61.3
C3	28772746	89.79	10.21	69.78
T1	22991940	78.55	21.45	67.72
T2	23065238	85.58	14.42	69.62
T3	26663626	88.02	11.98	76.75

C: control group; T: treated group with 0.8 mg/mL Z102-E.

Table S3. GO annotation results of *Listeria monocytogenes* DEGs

GO Category Number	Gene Number of BP	Gene Number of CC	Gene Number of MF	Gene Number	Percent of All Genes （%）
3	147	526	223	331	11.29

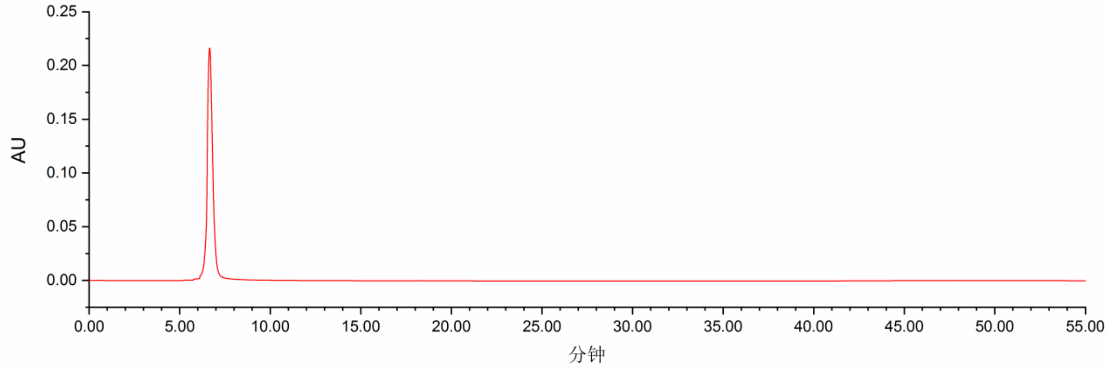

Figure S1: HPLC purification chromatogram of Z102-E.

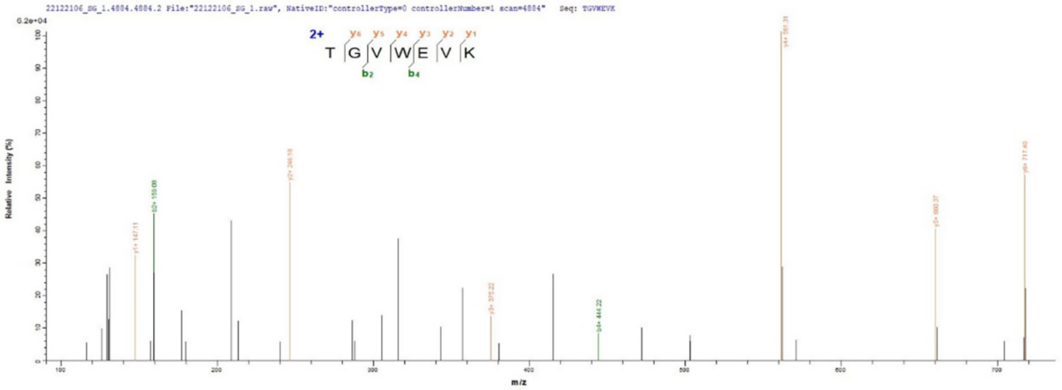

Figure S2: LC-MS/MS analysis of Z102-E.

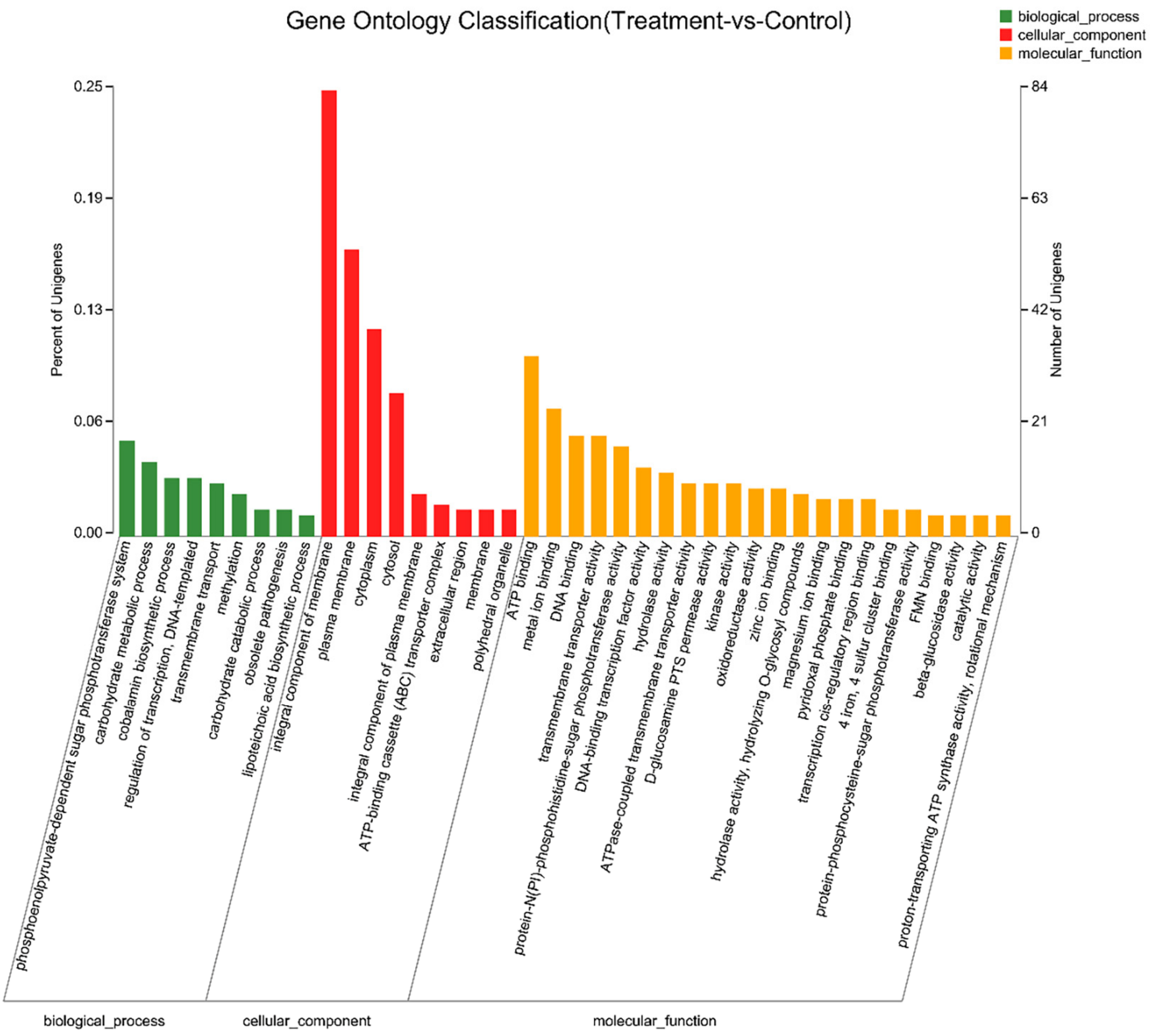

Figure S3. GO categorization. Unigene represents single gene

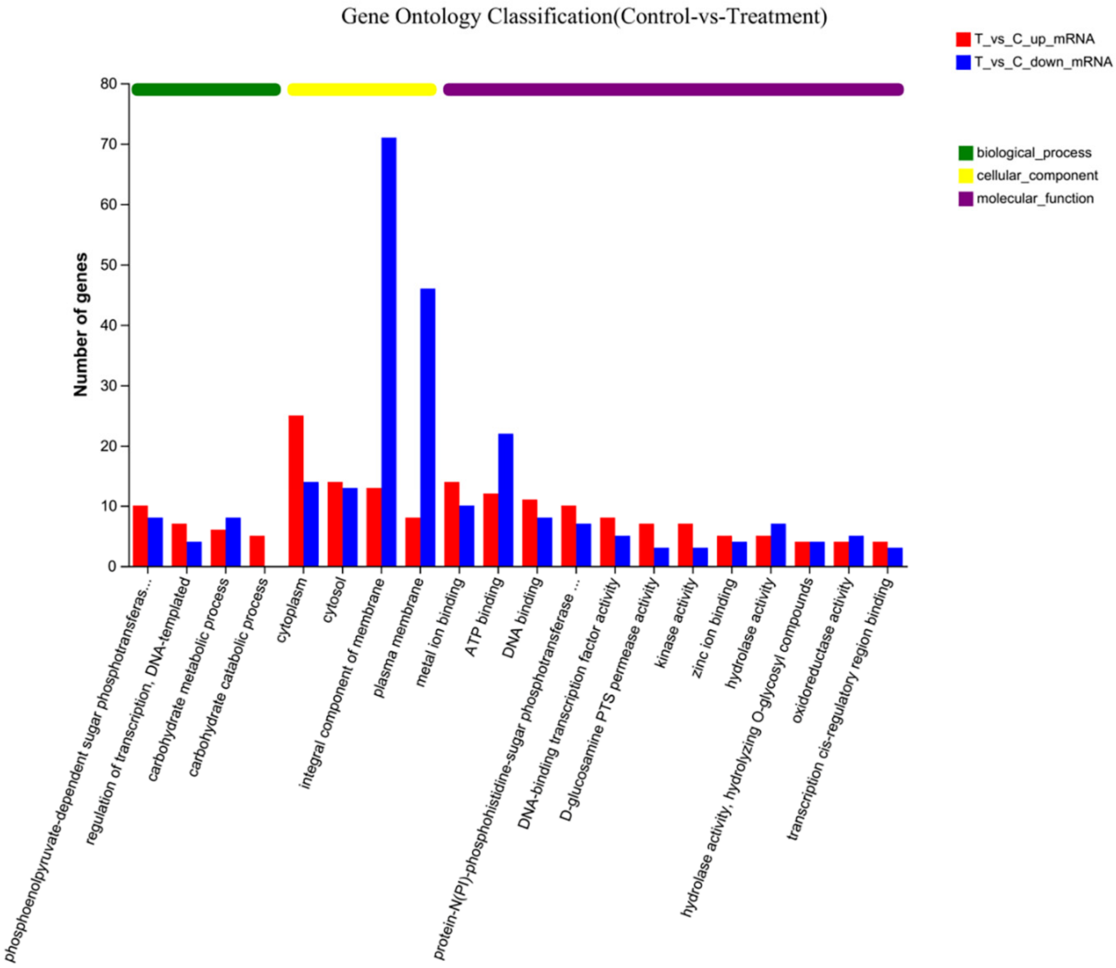

Figure S4. Classification of up-regulated and down-regulated genes in GO.

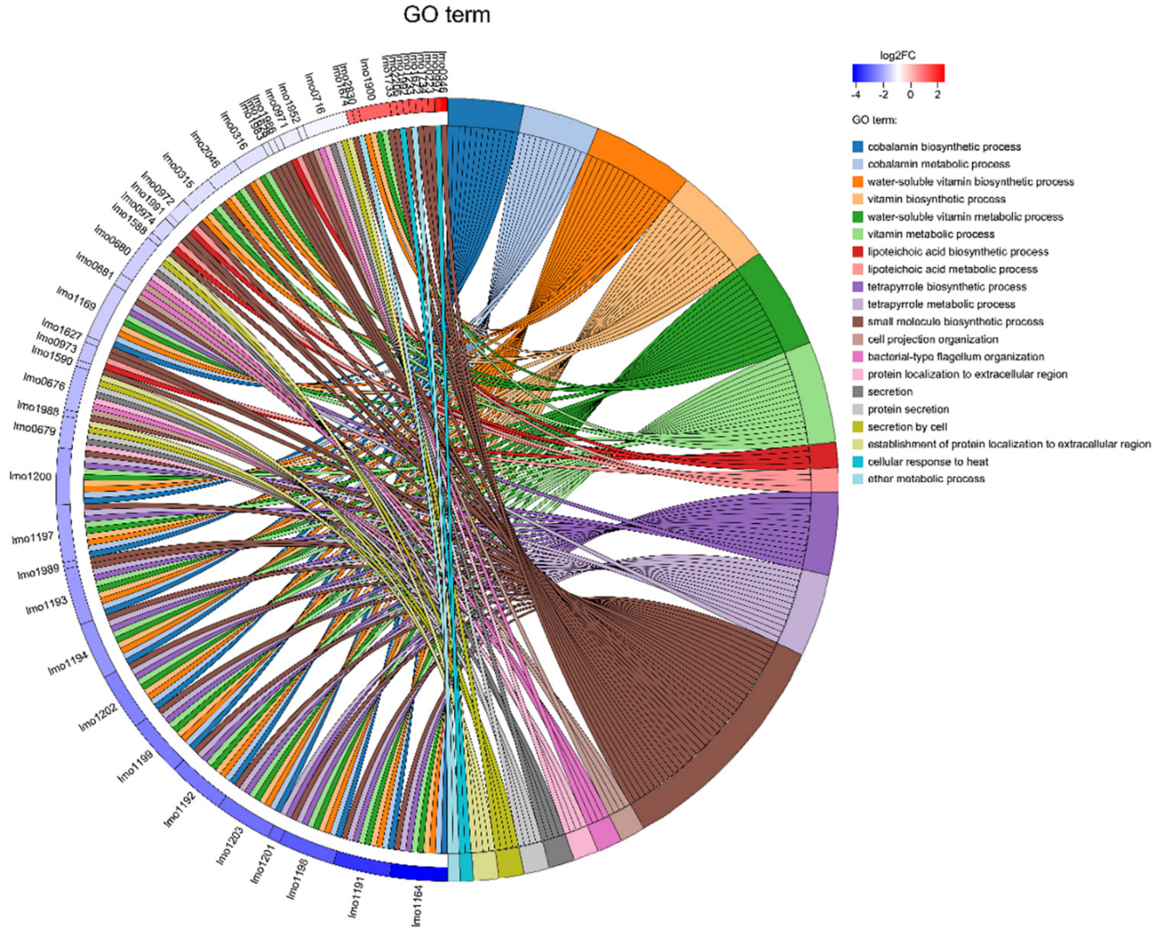

Figure S5. GO enrichment analysis.

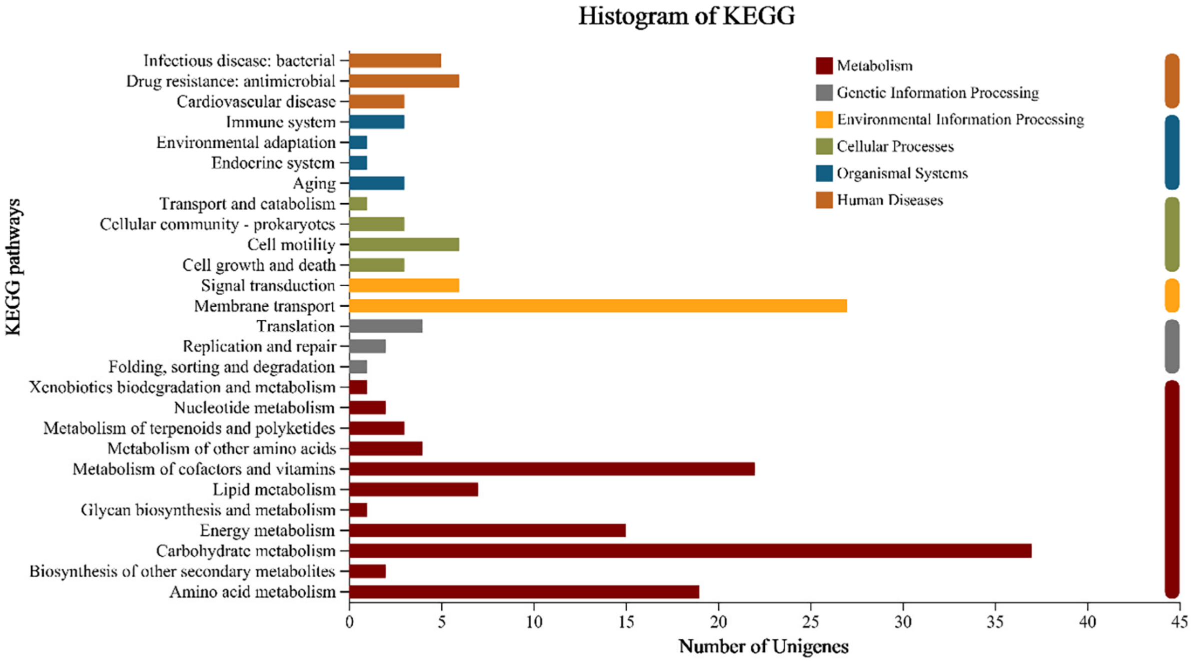

Figure S6. KEGG pathway classification.

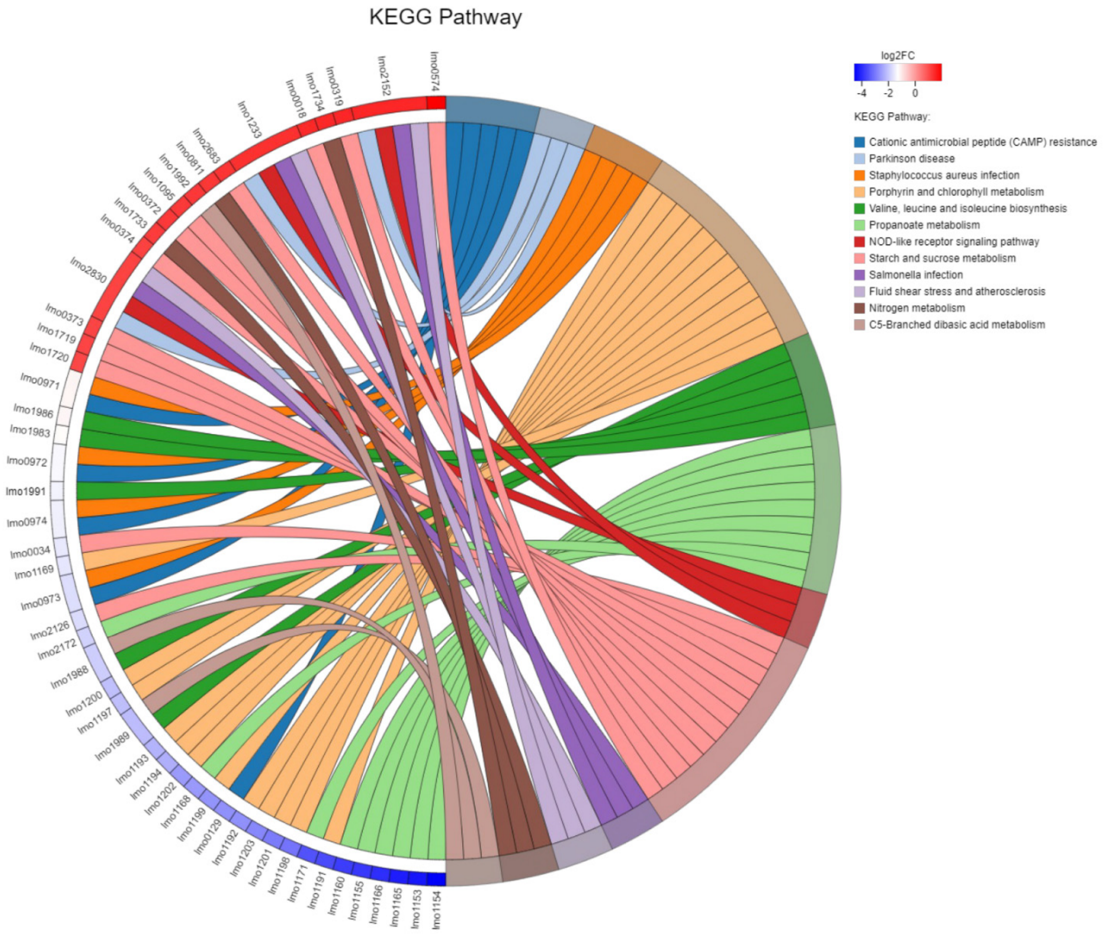

Figure S7. KEGG enrichment analysis.
